# Time to response with ravulizumab, a long‐acting terminal complement inhibitor, in adults with anti‐acetylcholine receptor antibody‐positive generalized myasthenia gravis

**DOI:** 10.1111/ene.16490

**Published:** 2024-10-07

**Authors:** Ali A. Habib, Michael Benatar, Tuan Vu, Andreas Meisel, Shahram Attarian, Masahisa Katsuno, Serena Liao, Kathleen N. Beasley, James F. Howard

**Affiliations:** ^1^ University of California Irvine California USA; ^2^ Department of Neurology University of Miami Miller School of Medicine Miami Florida USA; ^3^ Department of Neurology University of South Florida Morsani College of Medicine Tampa Florida USA; ^4^ Department of Neurology Charité—Universitätsmedizin Berlin Berlin Germany; ^5^ Reference Center for Neuromuscular Disorders and Amyotrophic Lateral Sclerosis, CHU La Timone Aix‐Marseille Université Marseille France; ^6^ Department of Neurology Nagoya University Graduate School of Medicine Nagoya Japan; ^7^ Alexion, AstraZeneca Rare Disease Boston Massachusetts USA; ^8^ Department of Neurology The University of North Carolina Chapel Hill North Carolina USA

**Keywords:** activities of daily living, complement inactivating agents, muscle strength, myasthenia gravis, generalized

## Abstract

**Background and Purpose:**

The efficacy and safety of ravulizumab, a terminal complement C5 inhibitor, in adults with anti‐acetylcholine receptor antibody‐positive (AChR Ab+) generalized myasthenia gravis (gMG) were demonstrated in the CHAMPION MG study (NCT03920293). This analysis aimed to characterize the latency to onset of a clinically meaningful therapeutic effect for ravulizumab.

**Methods:**

Post hoc analysis of data collected for up to 60 weeks from CHAMPION MG was performed to assess the timing of response to ravulizumab. Response was analyzed based on reductions of ≥2 and ≥3 points (minimal clinically important differences [MCIDs]) in Myasthenia Gravis–Activities of Daily Living (MG‐ADL) and Quantitative Myasthenia Gravis (QMG) total scores, respectively, and on more rigorous reductions of ≥3 and ≥5 points, respectively. Time to first response was assessed using the Kaplan–Meier product‐limit method.

**Results:**

The median (95% confidence interval) time to first response was 2.1 (2.1–2.6) and 4.1 (2.3–10.0) weeks for reductions of ≥2 and ≥3 points in MG‐ADL total score, respectively (*n* = 139), and 4.1 (2.1–10.0) and 18.3 (11.0–33.4) weeks for reductions of ≥3 and ≥5 points in QMG total score, respectively (*n* = 134). Cumulative response rates at Week 60 (data cut‐off) were 88% and 82% for ≥2‐ and ≥3‐point MG‐ADL score reductions, respectively, and 86% and 59% for ≥3‐ and ≥5‐point QMG score reductions, respectively.

**Conclusions:**

The median times to MCID with ravulizumab treatment in patients with AChR Ab+ gMG were ~2 weeks and ~4 weeks based on MCID MG‐ADL and QMG total score reductions, respectively.

## INTRODUCTION

Advances in our understanding of myasthenia gravis (MG) pathophysiology have aided the development of targeted treatment approaches with the potential to provide faster therapeutic benefit than mainstay treatments such as non‐steroidal immunosuppressive therapies (NSISTs), without the problematic side effects associated with broad immunosuppression. In particular, terminal complement inhibitors have been developed based on the importance of complement‐mediated effects on neuromuscular function in anti‐acetylcholine receptor antibody‐positive (AChR Ab+) MG (which accounts for ~85% patients with generalized MG [gMG]). Binding of a subset of these autoantibodies leads to activation of the classical complement cascade, which results in the architectural destruction of the postsynaptic membrane of the neuromuscular junction [1, 2, 3, 4, 5, 6].

Ravulizumab is a terminal complement C5 inhibitor that has been engineered to maintain therapeutic serum concentrations with an 8‐week dosing interval [7]. Ravulizumab was developed by modifying the humanized monoclonal antibody eculizumab with four specific amino acid substitutions that lead to reduced target‐mediated drug disposition (by increasing dissociation of the antibody from C5 in the endosome) and enhanced neonatal Fc receptor‐mediated recycling of the unbound antibody [7]. As a result, these modifications extend the elimination half‐life of the molecule and hence its duration of action [7]. The efficacy and safety of ravulizumab in patients with AChR Ab+ gMG were evaluated in CHAMPION MG, a 26‐week randomized, placebo‐controlled trial with an open‐label extension (OLE). The results demonstrated that ravulizumab treatment was associated with sustained symptom improvement, with a least‐squares mean (95% confidence interval [CI]) change from baseline of −3.1 (−3.8 to −2.3) in Myasthenia Gravis–Activities of Daily Living (MG‐ADL) total score and − 2.8 (−3.7 to −1.9) in Quantitative Myasthenia Gravis (QMG) total score at Week 26, compared with −1.4 (−2.1 to −0.7) and −0.8 (−1.7 to 0.1), respectively, in the placebo arm [8]. The interim findings from the OLE also support the long‐term efficacy and safety of ravulizumab [9].

The aim of the current post hoc analyses was to assess the timing of response to ravulizumab in terms of patients' functional abilities and muscle strength using data from the CHAMPION MG study. For the main post hoc analysis, we used the generally accepted thresholds for minimal clinically important difference (MCID) for improvement of a 2‐point reduction in MG‐ADL total score [10] and a 3‐point reduction in QMG total score [11]. To moderate some of the placebo effect and provide additional evidence to aid treatment decisions, analysis of timing of treatment response was also performed using more stringent thresholds (3‐ and 5‐point reductions in MG‐ADL and QMG total scores, respectively). Data based on both sets of thresholds are presented.

## METHODS

### CHAMPION MG

Full details of the methodology for CHAMPION MG (NCT03920293), including ethics approval and participant consent, have been reported previously [8, 9]. Briefly, patients were eligible for inclusion if they were aged ≥18 years and had AChR Ab+ gMG, a Myasthenia Gravis Foundation of America (MGFA) clinical classification of class II–IV, and an MG‐ADL total score ≥6. Patients were also required to have been vaccinated against meningococcal infections in the previous 3 years per local standards. Patients received ravulizumab (body‐weight‐based loading dose of 2400, 2700, or 3000 mg, followed by 3000, 3300, or 3600 mg at Week 2, then every 8 weeks thereafter) or placebo up to Week 26. At the end of the randomized controlled period (RCP) patients could enter the OLE and receive ravulizumab for up to 4 years. The blind was maintained at entry to the OLE, with both patients and investigators remaining unaware of the treatment received during the RCP. At Week 26, patients switching from placebo received a body‐weight‐based loading dose of ravulizumab as in the RCP, while patients who had received ravulizumab during the RCP were administered 900 mg to ensure maintenance of C5 inhibition until the next scheduled maintenance dose. For the next scheduled maintenance dose at Week 28 and every 8 weeks thereafter for up to 4 years, all patients received body‐weight‐based doses of ravulizumab 3000, 3300, or 3600 mg. Stable‐dose immunosuppressive therapies (including oral glucocorticoids) or acetylcholinesterase inhibitors were permitted throughout the RCP; dose changes of these agents were permitted during the OLE at the investigator's discretion.

The current interim analysis included data collected for up to 60 weeks from the RCP baseline in patients treated with ravulizumab (data cut‐off, 9 November 2021), irrespective of whether they received ravulizumab during the double‐blind RCP and OLE or just the OLE.

### Time to response analysis

Patients were eligible for inclusion in this post hoc analysis (response‐analysis population) if they initiated ravulizumab at the start of the RCP (MG‐ADL total score ≥6 was a requirement for entry) or if they had an MG‐ADL total score ≥6 at the start of the OLE for those who switched to ravulizumab. Time to response was assessed based on achieving a pre‐defined reduction from baseline in MG‐ADL or QMG total score during CHAMPION MG.

The MG‐ADL scale is a validated eight‐item, patient‐reported outcome measure that reflects ocular, bulbar, respiratory, and limb symptoms and their impact on function [12, 13]. Each item is graded on a 4‐point severity scale (from 0 = normal to 3 = most severe), with the total score ranging from 0 to 24; higher scores indicate greater functional impairment and disability. MG‐ADL response was analyzed based on the established definition of MCID in MG‐ADL of ≥2 points [10]; an analysis was also conducted using a more stringent definition of a reduction in total score of ≥3 points.

The QMG is a 13‐item, clinician‐reported scale that evaluates muscle strength based on the quantitative testing of sentinel muscle groups: ocular, facial, bulbar, gross motor, axial, and respiratory [14, 15]. All items are scored on a scale of 0–3 and the total score ranges from 0 to 39; higher scores indicate greater disease severity. QMG response was analyzed using the accepted (less stringent) definition of MCID in QMG of ≥3 points [11]; an analysis was also conducted based on the more conservative definition of a reduction in total score of ≥5 points. Permission to use the MG‐ADL questionnaire and the QMG form was obtained from Mapi Research Trust, Lyon, France, https://eprovide.mapi‐trust.org.

### Statistical analysis

Time to first response after ravulizumab initiation was assessed using the Kaplan–Meier product‐limit method. Response rates and cumulative response rates were determined at Weeks 1, 2, 4, 10, 12, 18, and 26, and at data cut‐off; there was no imputation for missing data. The cumulative response rate was conservatively calculated using the total sample size (*N* = 139) as the denominator for all study visits. Patient and clinical characteristics at baseline were summarized according to early or late response to ravulizumab (defined according to the median time to response determined using the Kaplan–Meier product‐limit method above) and those not meeting the response thresholds used in the study, to determine whether any of these characteristics might be predictive of response. Analyses were descriptive only; significance testing was not performed due to the small sample size of some of the subgroups derived as described above.

## RESULTS

### Study population

**FIGURE 1:**
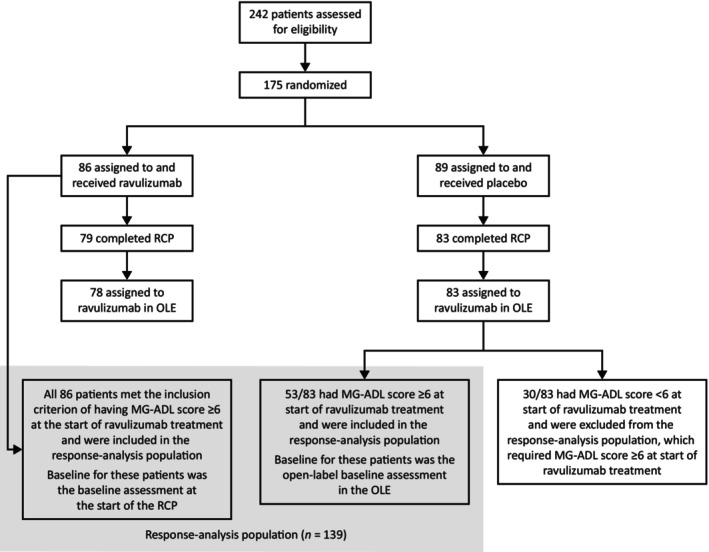
Patient populations for the CHAMPION randomized controlled period (RCP), open‐label extension (OLE), and response analysis. MG‐ADL, Myasthenia Gravis–Activities of Daily Living.

The response‐analysis population comprised 139 patients treated with ravulizumab who had an MG‐ADL total score ≥6 at the time of ravulizumab initiation (Figure 1). Five of these patients did not have QMG results available at data cut‐off; QMG results were therefore based on 134 patients with data available. The median (range) duration of ravulizumab treatment was 53.7 (2.0–63.1) weeks in the overall response‐analysis population, 60.1 (2.0–63.1) weeks in patients receiving ravulizumab in the RCP and OLE, and 34.0 (9.0–36.9) weeks in those who switched from placebo to ravulizumab at the start of the OLE.

### MG‐ADL responses

**FIGURE 2:**
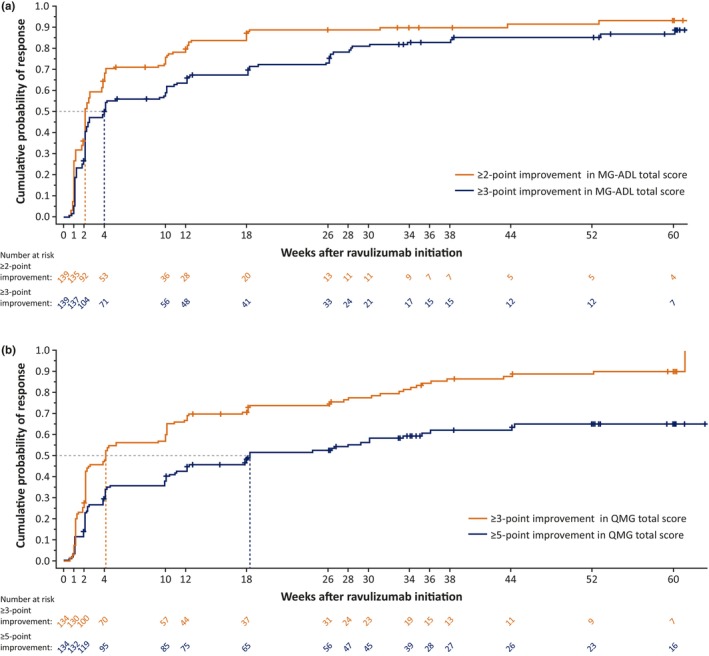
Cumulative probability of response to ravulizumab based on (a) reduction in Myasthenia Gravis–Activities of Daily Living (MG‐ADL) total score and (b) reduction in Quantitative Myasthenia Gravis (QMG) total score. Results from 139 patients with MG‐ADL data and 134 patients with QMG data available at cut‐off.

**FIGURE 3:**
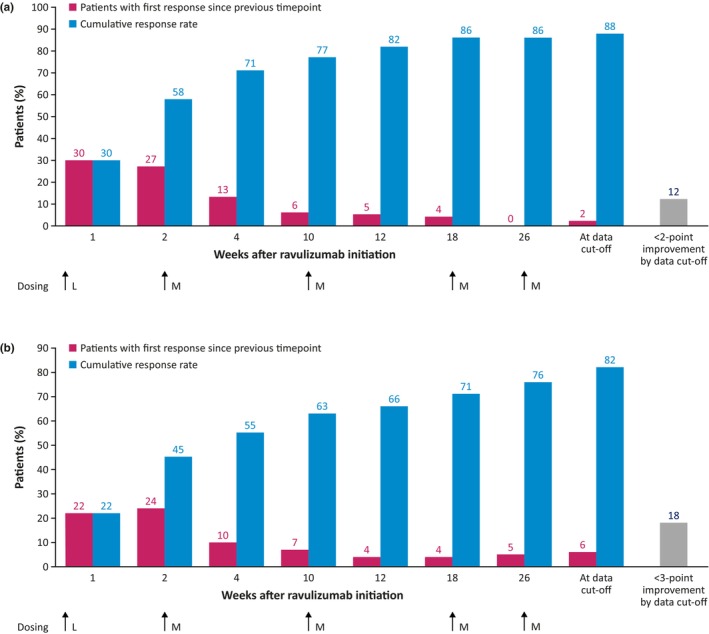
Response rates for ravulizumab over time according to (a) a ≥2‐point reduction and (b) a ≥3‐point reduction in Myasthenia Gravis–Activities of Daily Living total score. Cumulative data may not sum due to rounding. L, loading dose; M, maintenance dose.

Using the Kaplan–Meier analysis of response based on a definition of ≥2‐point reduction (MCID) in total MG‐ADL score, the median (95% CI) time to first MG‐ADL response in patients receiving ravulizumab in the RCP or OLE was 2.1 (2.1–2.6) weeks (Figure 2a). MG‐ADL early and late responders were therefore defined as those with a ≥2‐point reduction in MG‐ADL in ≤2 weeks and >2 weeks, respectively. The cumulative MG‐ADL response rate was 58% after 2 weeks' treatment, 86% after 26 weeks, and 88% at data cut‐off (Figure 3a). The 75th percentile value (95% CI) of the Kaplan–Meier estimate was 10.1 (4.1–12.4) weeks. The median (range) duration of ravulizumab treatment at data cut‐off was 54.3 (12.1–63.1) weeks in responders (*n* = 123) and 34.2 (2.0–61.1) weeks in patients not meeting the response threshold of 2‐point reduction in MG‐ADL total score (*n* = 16).

There were numerical differences in some baseline characteristics between early (≤2 weeks) and late (>2 weeks) MG‐ADL responders to ravulizumab, and patients not meeting the response threshold of a 2‐point reduction in MG‐ADL total score (Table 1). Compared with the early‐responder group and the group not meeting the response threshold, the late‐responder group included higher proportions of men and patients who had an MG crisis before study entry, and the patients had a longer median time from MG diagnosis to first ravulizumab dose. In the group not meeting the response threshold, the median MG‐ADL score at baseline was lower than in the responder groups.

**TABLE 1:** Baseline characteristics in response groups based on a ≥2‐point reduction in MG‐ADL total score.

Variable	Early responders^a^ (*n* = 80)	Late Responders^a^ (*n* = 43)	Patients not meeting the response threshold^b^, ^c^ (*n* = 16)
Male, *n* (%)	36 (45)	23 (53)	7 (44)
Age at first ravulizumab dose, years			
Mean (SD)	55.1 (14.9)	57.6 (15.9)	61.3 (16.2)
Median (min, max)	58.5 (19, 78)	60.0 (22, 82)	68.5 (30, 80)
Time from MG diagnosis at first ravulizumab dose, years			
Mean (SD)	10.2 (9.9)	10.5 (9.5)	9.1 (7.8)
Median (min, max)	6.1 (0.6, 39.5)	9.2 (1.1, 36.6)	5.1 (1.9, 28.2)
Baseline MG‐ADL total score^d^			
Mean (SD)	9.6 (2.4)	8.8 (2.7)	8.6 (4.5)
Median (min, max)	9.0 (6, 19)	8.0 (6, 17)	7.0 (6, 24)
MGFA disease class at screening, *n* (%)			
IIa	17 (21)	12 (28)	5 (31)
IIb	16 (20)	7 (16)	2 (13)
IIIa	22 (28)	13 (30)	5 (31)
IIIb	19 (24)	6 (14)	2 (13)
IVa	2 (3)	4 (9)	0
IVb	3 (4)	1 (2)	2 (13)
Missing	1 (1)	0	0
ISTs before study entry^e^, ^f^ *n* (%)			
0	5 (6)	1 (2)	0
1	27 (34)	14 (33)	5 (31)
2	40 (50)	22 (51)	8 (50)
3	8 (10)	6 (14)	3 (19)
MG exacerbation before study entry, *n* (%)	52 (65)	26 (60)	8 (50)
MG crisis before study entry, *n* (%)	17 (21)	13 (30)	3 (19)

Abbreviations: IST, immunosuppressant therapy; MG, myasthenia gravis; MG‐ADL, Myasthenia Gravis–Activities of Daily Living; MGFA, Myasthenia Gravis Foundation of America; SD, standard deviation.

^a^ Early and late MG‐ADL response was defined as reduction in MG‐ADL total score ≥2 points within (early) and after (late) 2 weeks of ravulizumab treatment, respectively.

^b^ At the time of the interim analysis.

^c^ Patients not meeting the response threshold were defined as those who had a change in score from baseline that was less than the specified threshold improvement at data cut‐off or Week 60, whichever was earlier.

^d^ The last available assessment value before the first ravulizumab infusion.

^e^ Medications taken within 2 years before informed consent and up to the first dose of study drug infusion.

^f^ Corticosteroids, azathioprine, mycophenolate mofetil, cyclosporin, tacrolimus, methotrexate, and/or cyclophosphamide.

Using the Kaplan–Meier analysis of response based on a ≥3‐point reduction in MG‐ADL total score, the median (95% CI) time to first MG‐ADL response in patients receiving ravulizumab in the RCP or OLE was estimated as 4.1 (2.3–10.0) weeks (Figure 2a). MG‐ADL early and late responders were therefore defined as those with a response at ≤4 and >4 weeks, respectively. The cumulative MG‐ADL response rate continued to increase at each timepoint at which it was assessed (Figure 3b). Cumulative response rates were 45% after 2 weeks' treatment (i.e., after the first ravulizumab dose), 76% after 26 weeks, and 82% at data cut‐off. The 75th percentile value (95% CI) of the Kaplan–Meier estimate was 26.1 (18.1–33.9) weeks. The median (range) duration of ravulizumab treatment at data cut‐off was 54.5 (12.1–63.1) weeks in responders (*n* = 114) and 34.3 (2.0–61.1) weeks in patients not meeting the response threshold of a 3‐point reduction in MG‐ADL total score (*n* = 25).

Baseline characteristics in the different response groups are summarized in Table 2. Similar patterns of between‐group differences were observed as for the groups defined by the less stringent MG‐ADL score.

**TABLE 2:** Baseline characteristics in response groups based on a ≥3‐point reduction in MG‐ADL total score.

Variable	Early responders^a^ (*n* = 77)	Late responders^a^ (*n* = 37)	Patients not meeting the response threshold^b^, ^c^ (*n* = 25)
Male, *n* (%)	35 (45)	21 (57)	10 (40)
Age at first ravulizumab dose, years			
Mean (SD)	55.1 (14.0)	57.7 (17.7)	59.5 (15.6)
Median (min, max)	57.0 (19, 78)	63.0 (21, 82)	66.0 (30, 80)
Time from MG diagnosis at first ravulizumab dose, years			
Mean (SD)	9.2 (9.5)	12.6 (10.5)	9.3 (7.2)
Median (min, max)	5.3 (0.6, 36.6)	9.4 (1.1, 39.5)	6.5 (1.9, 28.2)
Baseline MG‐ADL total score^d^			
Mean (SD)	9.7 (2.7)	8.8 (2.2)	8.4 (3.8)
Median (min, max)	10.0 (6, 19)	9.0 (6, 14)	7.0 (6, 24)
MGFA disease class at screening, *n* (%)			
IIa	19 (25)	9 (24)	6 (24)
IIb	15 (19)	6 (16)	4 (16)
IIIa	20 (26)	12 (32)	8 (32)
IIIb	17 (22)	6 (16)	4 (16)
IVa	2 (3)	3 (8)	1 (4)
IVb	3 (4)	1 (3)	2 (8)
Missing	1 (1)	0	0
ISTs before study entry^e^, ^f^ *n* (%)			
0	4 (5)	1 (3)	1 (4)
1	23 (30)	14 (38)	9 (36)
2	40 (52)	18 (49)	12 (48)
3	10 (13)	4 (11)	3 (12)
MG exacerbation before study entry, *n* (%)	50 (65)	23 (62)	13 (52)
MG crisis before study entry, *n* (%)	14 (18)	13 (35)	6 (24)

Abbreviations: IST, immunosuppressant therapy; MG, myasthenia gravis; MG‐ADL, Myasthenia Gravis–Activities of Daily Living; MGFA, Myasthenia Gravis Foundation of America; SD, standard deviation.

^a^ Early and late MG‐ADL response was defined as reduction in MG‐ADL total score ≥3 points within (early) and after (late) 4 weeks of ravulizumab treatment, respectively.

^b^ At the time of the interim analysis.

^c^ Patients not meeting the response threshold were defined as those who had a change in score from baseline that was less than the specified threshold improvement at data cut‐off or Week 60, whichever was earlier.

^d^ The last available assessment value before the first ravulizumab infusion.

^e^ Medications taken within 2 years before informed consent and up to the first dose of study drug infusion.

^f^ Corticosteroids, azathioprine, mycophenolate mofetil, cyclosporin, tacrolimus, methotrexate, and/or cyclophosphamide.

### QMG responses

**FIGURE 4:**
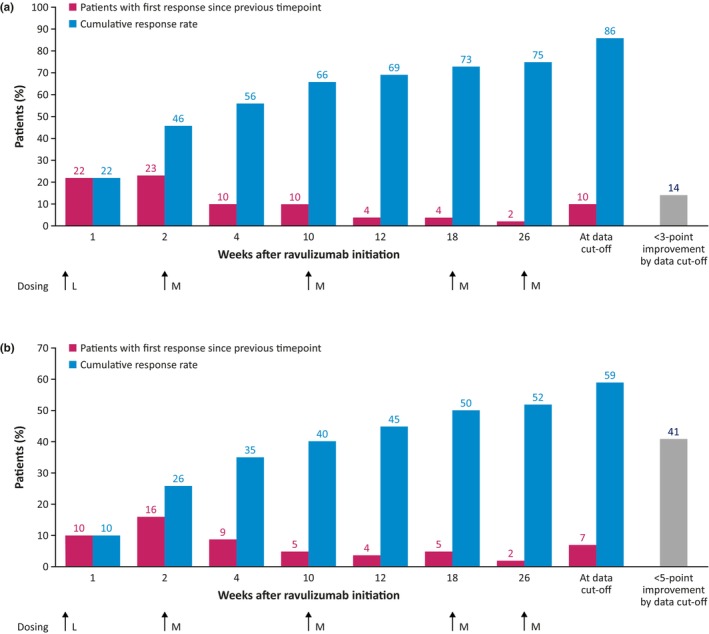
Response rates for ravulizumab over time according to (a) a ≥3‐point reduction and (b) a ≥5‐point reduction in Quantitative Myasthenia Gravis total score. Cumulative data may not sum due to rounding. L, loading dose; M, maintenance dose.

For the 134 patients with QMG data available, the median (95% CI) time to first QMG response based on a ≥3‐point reduction (MCID) in total QMG score in patients receiving ravulizumab in the RCP or OLE was 4.1 (2.1–10.0) weeks (Figure 2b). QMG early and late responders were therefore defined as those with a response in ≤4 weeks and >4 weeks, respectively. The cumulative QMG response rates were 46% after 2 weeks' treatment, 75% after 26 weeks, and 86% at data cut‐off (Figure 4a). The 75th percentile value of the Kaplan–Meier estimate was 26.3 (95% CI 12.1–34.1) weeks. The median (range) duration of ravulizumab treatment at data cut‐off was 54.7 (9.0–63.1) weeks in responders (*n* = 115) and 35.1 (2.0–60.3) weeks in patients not meeting the response threshold of 3‐point reduction in QMG total score (*n* = 19).

There were numerical differences in some baseline characteristics between early (≤4 weeks) and late (>4 weeks) responders to ravulizumab and patients not meeting the response threshold (Table 3). Compared with the other response groups, in the early‐responder group, mean baseline QMG scores were higher, a greater proportion of patients had MG exacerbation before study entry, and a lower proportion had an MG crisis before study entry. In the late‐responder group, more patients had MGFA Class IIa disease and fewer had Class IIIb disease than in the other groups. The median time from MG diagnosis at ravulizumab initiation was longer in the group not meeting the response threshold than in the other groups.

**TABLE 3:** Baseline characteristics in response groups based on a ≥3‐point reduction in QMG total score.

Variable	Early responders^a^ (*n* = 75)	Late responders^a^ (*n* = 40)	Patients not meeting the response threshold^b^, ^c^ (*n* = 19)
Male, *n* (%)	35 (47)	22 (55)	7 (37)
Age at first ravulizumab dose, years			
Mean (SD)	56.6 (14.2)	58.4 (14.4)	56.5 (19.3)
Median (min, max)	58.0 (19, 82)	62.0 (30, 79)	62.0 (20, 80)
Time from MG diagnosis at first ravulizumab dose, years			
Mean (SD)	10.6 (10.6)	9.8 (8.9)	10.4 (7.0)
Median (min, max)	5.5 (0.6, 39.5)	7.5 (1.3, 36.6)	9.4 (1.1, 28.2)
Baseline QMG total score^d^			
Mean (SD)	16.2 (4.1)	13.3 (4.4)	14.6 (7.5)
Median (min, max)	17.0 (6, 26)	14.0 (6, 22)	12.0 (6, 39)
MGFA disease class at screening, *n* (%)			
IIa	15 (20)	15 (38)	3 (16)
IIb	12 (16)	6 (15)	6 (32)
IIIa	23 (31)	12 (30)	4 (21)
IIIb	18 (24)	4 (10)	4 (21)
IVa	2 (3)	3 (8)	0
IVb	4 (5)	0	2 (11)
Missing	1 (1)	0	0
ISTs before study entry,^e^, ^f^ *n* (%)			
0	4 (5)	2 (5)	0
1	27 (36)	10 (25)	8 (42)
2	34 (45)	23 (58)	10 (53)
3	10 (13)	5 (13)	1 (5)
MG exacerbation before study entry, *n* (%)	52 (69)	20 (50)	11 (58)
MG crisis before study entry, *n* (%)	14 (19)	13 (33)	6 (32)

Abbreviations: IST, immunosuppressant therapy; MG, myasthenia gravis; MGFA, Myasthenia Gravis Foundation of America; QMG, Quantitative Myasthenia Gravis; SD, standard deviation.

^a^ Early and late QMG response was defined as reduction in QMG total score ≥3 points within (early) and after (late) 4 weeks of ravulizumab treatment, respectively.

^b^ At the time of the interim analysis.

^c^ Patients not meeting the response threshold were defined as those who had a change in score from baseline that was less than the specified threshold improvement at data cut‐off or Week 60, whichever was earlier.

^d^ The last available assessment value before the first ravulizumab infusion.

^e^ Medications taken within 2 years before informed consent and up to the first dose of study drug infusion.

^f^ Corticosteroids, azathioprine, mycophenolate mofetil, cyclosporin, tacrolimus, methotrexate, and/or cyclophosphamide.

Using a more stringent ≥5‐point reduction in QMG total score as an analysis threshold, the median (95% CI) time to first QMG response in patients receiving ravulizumab in the RCP or OLE was 18.3 (11.0–33.4) weeks (Figure 2b). QMG early and late responders were therefore defined as those with a response in ≤18 weeks and >18 weeks, respectively. The cumulative QMG response rate continued to increase at each timepoint at which it was assessed (Figure 4b). Response rates were 26% after 2 weeks' treatment (after the first ravulizumab dose), 52% after 26 weeks, and 59% at data cut‐off. The 75th percentile value of the Kaplan–Meier estimate was not reached at data cut‐off. The median (range) duration of ravulizumab treatment at data cut‐off was 59.1 (21.9–61.1) weeks in responders (*n* = 79) and 35.1 (2.0–63.1) weeks in patients not meeting the response threshold of a 5‐point reduction in QMG total score (*n* = 55).

Baseline characteristics in the different response groups are summarized in Table 4; similar patterns of between‐group differences were observed as for the groups defined by the less stringent QMG score threshold.

**TABLE 4:** Baseline characteristics in response groups based on a ≥5‐point reduction in QMG total score.

	Early responders^a^ (*n* = 67)	Late responders^a^ (*n* = 12)	Patients not meeting the response threshold^b^, ^c^ (*n* = 55)
Male, *n* (%)	30 (45)	6 (50)	28 (51)
Age at first ravulizumab dose, years			
Mean (SD)	56.1 (14.1)	59.4 (11.5)	57.8 (16.7)
Median (min, max)	58.0 (19, 78)	58.5 (37, 79)	63.0 (20, 82)
Time from MG diagnosis at first ravulizumab dose, years			
Mean (SD)	10.0 (10.8)	9.1 (9.3)	11.0 (8.2)
Median (min, max)	5.0 (0.6, 39.5)	3.6 (1.3, 26.4)	9.2 (1.1, 36.6)
Baseline QMG total score^d^			
Mean (SD)	16.0 (4.3)	14.0 (4.7)	14.3 (5.5)
Median (min, max)	16.0 (6, 26)	15.5 (7, 20)	14.0 (6, 39)
MGFA disease class at screening, *n* (%)			
IIa	17 (25)	5 (42)	11 (20)
IIb	10 (15)	1 (8)	13 (24)
IIIa	17 (25)	4 (33)	18 (33)
IIIb	16 (24)	1 (8)	9 (16)
IVa	2 (3)	1 (8)	2 (4)
IVb	4 (6)	0	2 (4)
Missing	1 (1)	0	0
ISTs before study entry^e^, ^f^ *n* (%)			
0	4 (6)	0	2 (4)
1	24 (36)	3 (25)	18 (33)
2	30 (45)	7 (58)	30 (55)
3	9 (13)	2 (17)	5 (9)
MG exacerbation before study entry, *n* (%)	46 (69)	5 (42)	32 (58)
MG crisis before study entry, *n* (%)	10 (15)	3 (25)	20 (36)

Abbreviations: IST, immunosuppressant therapy; MG, myasthenia gravis; MGFA, Myasthenia Gravis Foundation of America; QMG, Quantitative Myasthenia Gravis; SD, standard deviation.

^a^ Early and late QMG response was defined as reduction in QMG total score ≥5 points within (early) and after (late) 18 weeks of ravulizumab treatment, respectively.

^b^ At the time of the interim analysis.

^c^ Patients not meeting the response threshold were defined as those who had a change in score from baseline that was less than the specified threshold improvement at data cut‐off or Week 60, whichever was earlier.

^d^ The last available assessment value before the first ravulizumab infusion.

^e^ Medications taken within 2 years before informed consent and up to the first dose of study drug infusion.

^f^ Corticosteroids, azathioprine, mycophenolate mofetil, cyclosporin, tacrolimus, methotrexate, and/or cyclophosphamide.

## DISCUSSION

The current analysis assessed the time to response with ravulizumab treatment in patients with gMG, using data from the CHAMPION MG study. Using Kaplan–Meier analyses, the median time to first response was estimated to be approximately 2 weeks according to the MG‐ADL score and 4 weeks according to the QMG score, based on reductions generally accepted as the MCID for each measure [10, 11]. Assessments based on more stringent response thresholds gave longer estimated median times to first response of approximately 4 and 18 weeks for the MG‐ADL and QMG scores, respectively.

Consistent with findings for other C5 inhibitors [16, 17], the current analysis showed that many patients had a rapid response to ravulizumab treatment. The rapid onset—by Week 2 in many patients—differentiates ravulizumab from NSISTs, which typically demonstrate effectiveness only after several months of treatment [18, 19, 20]. This represents an important potential clinical benefit for patients; in particular, the rapid onset of improvement in the MG‐ADL score with ravulizumab treatment reflects the ability to quickly recover function in routine daily activities, from the patient's perspective. Plasma exchange therapy has an onset of action within a few days and intravenous immunoglobulin therapy within a slightly longer time period; these are usually administered short‐term only for the control of severe MG or acute exacerbations, although they are used for maintenance treatment in patients with refractory gMG [18, 21].

The results also indicate that the first response to ravulizumab may be slower in some patients, indicating that a longer “trial of therapy” may be required before it is considered ineffective. The reason for the delayed response in some patients is not clear. It may reflect cumulative effects of concomitant slower‐acting NSISTs, which were permitted during the study. A delayed response has also been observed in some eculizumab‐treated patients: in the phase 3 REGAIN trial, most patients had a clinical response by Week 12, but first responses were also observed with longer‐term treatment [16].

Another interesting observation is the difference in time to first response when assessed using the MG‐ADL and QMG scores, although the difference was much smaller when the less stringent MCID criteria were applied. This slower QMG response was also observed in an analysis of time to first response for eculizumab in patients with refractory, anti‐AChR Ab+ gMG [22]. Such disparate timing may derive from differences in the cut‐off scores used to define response. Another possible explanation is that the MG‐ADL assessment is more qualitative (and therefore more susceptible to a placebo effect), while the QMG assessment is more quantitative. It has previously been shown that the correlation between MG‐ADL and QMG scores (change from baseline post treatment) increases with time [23], suggesting that the MG‐ADL questionnaire is more sensitive to change than the QMG scale. In clinical practice, the results of the current analysis suggest that patients may perceive improvements in their disease as early as 2 weeks after starting ravulizumab, but that objective physician‐rated improvements in muscle weakness may take longer. Thus, if physicians use QMG rather than MG‐ADL scores to assess patients in clinical practice, a longer “trial of therapy” may be required.

Data from the current analysis may also help inform decisions on the appropriate duration of a trial of ravulizumab treatment for patients who do not experience a rapid improvement in symptoms. At the data cut‐off, 12% and 18% of patients did not meet the 2‐ and 3‐point response thresholds, respectively, in MG‐ADL total score, and 14% and 41% did not meet the 3‐ and 5‐point response thresholds, respectively, in QMG total score. Some (but not all) of these patients may continue to derive benefit from longer treatment, although the requirement for continued treatment needs to be weighed against cost and other potential therapeutic strategies that might be employed. It is possible that an analysis of the characteristics of patients with different timing of response could provide insights into the appropriate duration of a trial of ravulizumab treatment. Analysis of the demographic and clinical characteristics of early responders, late responders, and patients not meeting the response threshold in CHAMPION MG provides some interesting observations. However, based on descriptive analysis, there were no clear trends and the data are not sufficiently robust to draw firm conclusions relevant to predicting the timing of patients' response to ravulizumab. This may reflect the small patient numbers (and hence high interpatient variability), and further work is needed to identify patients most likely to respond to ravulizumab and to determine how long to continue therapy in those who do not respond quickly. As well as time to onset of therapeutic effect, factors to be considered when selecting treatment for gMG include comparative effectiveness, safety and tolerability, patient convenience, and cost.

Limitations of the current analysis include its post hoc nature and the low patient numbers in some of the response groups. The absence of a comparison against placebo through 60 weeks of treatment also complicates interpretation of the results, although analysis of the ravulizumab and placebo study arms during the 26‐week RCP demonstrated a significantly shorter median time to MG‐ADL and QMG response for ravulizumab compared with placebo (Tables S1 and S2). A considerable placebo effect was observed in the MG‐ADL response data (and to a lesser extent in the QMG response data) in the RCP. This was also noted in the primary analyses of clinical outcomes from the CHAMPION MG study [8]. Although the cause of the placebo effect is unknown, this is consistent with findings in other phase 2 and 3 clinical studies in gMG [22, 24, 25, 26]. An additional limitation is the interim nature of the analysis, which meant that there was limited follow‐up for some patients.

In conclusion, a considerable proportion of patients had a first response within 2 weeks of initiating ravulizumab treatment, even when assessed using thresholds above MCIDs. The results also suggest that, when using ravulizumab in clinical practice, a longer than anticipated treatment trial may be warranted for some patients before considering treatment discontinuation. To obtain a fuller clinical picture when evaluating treatment effectiveness, MG‐ADL scores and, if possible, QMG scores should be compared with those before treatment initiation, and cumulative changes should be assessed over time. Further work to evaluate patient and disease characteristics that predict response and timing of response would be beneficial.

## AUTHOR CONTRIBUTIONS

**Ali A. Habib:** Investigation; writing – review and editing; resources. **Michael Benatar:** Conceptualization; methodology; resources; writing – review and editing. **Tuan Vu:** Investigation; resources; writing – review and editing. **Andreas Meisel:** Investigation; resources; writing – review and editing. **Shahram Attarian:** Investigation; resources; visualization; validation; writing – review and editing. **Masahisa Katsuno:** Resources; writing – review and editing. **Serena Liao:** Methodology; formal analysis; software; writing – review and editing. **Kathleen N. Beasley:** Conceptualization; methodology; supervision; writing – review and editing. **James F. Howard Jr:** Investigation; resources; writing – review and editing.

## FUNDING INFORMATION

This study was funded by Alexion, AstraZeneca Rare Disease.

## CONFLICT OF INTEREST STATEMENT

**Ali A. Habib** has served as a medical advisor and speaker on behalf of Alexion, AstraZeneca Rare Disease, has received research support from Alexion, AstraZeneca Rare Disease, and has received research support and honoraria from argenx, UCB, Immunovant, Cabaletta Bio, Pfizer, Genentech/Roche, Viela Bio/Horizon, and Regeneron. **Michael Benatar** reports consulting for Alector, Alexion, Annexon, Arrowhead, Biogen, Cartesian, Denali, Eli Lilly, Horizon, Immunovant, Janssen, Novartis, Roche, Sanofi, Takeda, UCB, and uniQure. **Tuan Vu** is the USF Site Principal Investigator for MG clinical trials sponsored by Alexion, AstraZeneca Rare Disease, argenx, UCB Pharma, Amgen, Johnson & Johnson, Immunovant, Dianthus, Sanofi, Regeneron, and Cartesian Therapeutics, and receives speaker and/or consultant honoraria from Alexion, AstraZeneca Rare Disease, UCB, Dianthus, ImmunAbs, and argenx. **Andreas Meisel** has received speaker honoraria, consulting fees, or financial research support (paid to institution) from Alexion, AstraZeneca Rare Disease, argenx, Axunio, Grifols, Hormosan, Janssen, Merck, Octapharma, and UCB. He serves as chairman of the medical advisory board of the German Myasthenia Gravis Society. **Shahram Attarian** has received honoraria from Biogen, Roche, Sanofi, Pfizer, Alnylam, argenx, LFB, and Pharnext, and has received research support (paid to institution) from Pfizer, Biogen, and LFB. **Masahisa Katsuno** has received speaker honoraria from Biogen Japan, Chugai, and Eisai, and financial research support from Alexion, AstraZeneca Rare Disease, argenx, Eisai, and Mitsubishi‐Tanabe. **Serena Liao** is an employee of Alexion, AstraZeneca Rare Disease and owns stock in AstraZeneca. **Kathleen N. Beasley** was an employee of Alexion, AstraZeneca Rare Disease and owned stock in AstraZeneca at the time the study was conducted. **James F. Howard Jr** has received research support (paid to institution) from Ad Scientiam, argenx, Cartesian Therapeutics, the Centers for Disease Control and Prevention (Atlanta, GA, USA), the Myasthenia Gravis Foundation of America, the Muscular Dystrophy Association, the National Institutes of Health (including the National Institute of Neurological Disorders and Stroke and the National Institute of Arthritis and Musculoskeletal and Skin Diseases), PCORI, and UCB Bioscience, honoraria from Ad Scientiam, AcademicCME, Alexion, AstraZeneca Rare Disease, argenx, Biohaven Ltd., Biologix Pharma, CheckRare CME, F. Hoffman‐LaRoche Ltd., Horizon Therapeutics plc (now Amgen), Medscape CME, Merck EMB Serono, Novartis Pharmaceuticals, PeerView CME, Physicians' Education Resource (PER) CME, PlatformQ CME, Regeneron Pharmaceuticals, Sanofi US, UCB Biosciences, and Zai Laboratories, and non‐financial support from Alexion, AstraZeneca Rare Disease, argenx, Cartesian Therapeutics, Toleranzia AB, UCB Biosciences, and Zai Laboratories.

## Supporting information


Data S1:

## Data Availability

Alexion, AstraZeneca Rare Disease will consider requests for disclosure of clinical study participant‐level data provided that participant privacy is assured through methods such as data de‐identification, pseudonymization, or anonymization (as required by applicable law), and if such disclosure was included in the relevant study informed consent form or similar documentation. Qualified academic investigators may request participant‐level clinical data and supporting documents (statistical analysis plan and protocol) pertaining to Alexion‐sponsored studies. Further details regarding data availability and instructions for requesting information are available in the Alexion Clinical Trials Disclosure and Transparency Policy at https://www.alexionclinicaltrialtransparency.com/data‐requests/.
