# Supplementary material for: Time to response with ravulizumab, a long‐acting terminal complement inhibitor, in adults with anti‐acetylcholine receptor antibody‐positive generalized myasthenia gravis
Source: Eur J Neurol. 2024 Oct 7;31(12):e16490. doi: 10.1111/ene.16490 (PMC11555155; doi:10.1111/ene.16490)
Supplement: Supplementary file 1 — Data S1: [file ENE-31-e16490-s001.docx]

**Time to response with ravulizumab, a long-acting terminal complement inhibitor, in adults with anti-acetylcholine receptor antibody-positive generalized myasthenia gravis**

Ali A Habib, Michael Benatar, Tuan Vu, Andreas Meisel, Shahram Attarian, Masahisa Katsuno, Serena Liao, Kathleen N Beasley, James F Howard Jr

**Supplementary information**

**Supplementary Table 1. Time to first MG-ADL response in patients receiving placebo or ravulizumab during the 26-week randomized controlled period of CHAMPION MG**

| **Time to first response** | **≥2-point MG-ADL total score reduction** | | **≥3-point MG-ADL total score reduction** | |
| --- | --- | --- | --- | --- |
|  | **Placebo** | **Ravulizumab** | **Placebo** | **Ravulizumab** |
| KM estimate; all randomized, treated patients^a^ | n=89 | n=86 | n=89 | n=86 |
| 25^th^ percentile (95% CI), weeks | 1.1 (1.1–1.7) | 1.1 (NA–NA) | 2.1 (1.1–4.1) | 1.1 (1.1–1.3) |
| 50^th^ percentile (95% CI), weeks | 4.1 (2.1–10.1) | 1.9 (1.1–3.9) | 12.1 (10.0–26.0) | 4.1 (2.1–12.1) |
| 75^th^ percentile (95% CI), weeks | 18.4 (12.1–NA) | 10.4 (4.1–12.7) | NA | 26.2 (12.4–NA) |
| p-value^b^ vs placebo |  | 0.0251 |  | 0.0471 |
| Patients with response | n=69 | n=74 | n=54 | n=62 |
| Mean (SD), weeks | 5.4 (6.4) | 3.9 (4.8) | 6.5 (6.4) | 5.6 (7.3) |
| Median (min, max), weeks | 2.1 (1.0, 26.3) | 1.3 (0.9, 18.4) | 4.1 (1.0, 26.1) | 1.9 (0.9, 26.3) |

^a^Based on Kaplan–Meier product-limit method; ^b^Log-rank test.

CI, confidence interval; KM, Kaplan–Meier; MG-ADL, Myasthenia Gravis–Activities of Daily Living; NA, not available; SD, standard deviation.

**Supplementary Table 2. Time to first QMG response in patients receiving placebo or ravulizumab during the 26-week randomized controlled period of CHAMPION MG**

| **Time to first response** | **≥3-point QMG total score reduction** | | **≥5-point QMG total score reduction** | |
| --- | --- | --- | --- | --- |
|  | **Placebo** | **Ravulizumab** | **Placebo** | **Ravulizumab** |
| KM estimate; all randomized, treated patients^a^ | n=89 | n=86 | n=89 | n=86 |
| 25^th^ percentile (95% CI) | 2.1 (1.3–4.1) | 1.1 (1.1–1.4) | 18.1 (10.1–NA) | 2.1 (1.1–4.1) |
| 50^th^ percentile (95% CI) | 18.1 (10.3–NA) | 4.1 (2.1–10.0) | NA (26.3–NA) | 18.1 (10.0–NA) |
| 75^th^ percentile (95% CI) | NA | 26.3 (12.1–NA) | NA | 26.4 (26.4–NA) |
| p-value^b^ vs placebo |  | 0.0081 |  | 0.0005 |
| Patients with response | n=50 | n=61 | n=27 | n=47 |
| Mean (SD), weeks | 7.1 (7.5) | 4.2 (5.4) | 9.9 (7.8) | 6.3 (6.8) |
| Median (min, max), weeks | 3.0 (0.9, 26.1) | 1.9 (0.6, 26.3) | 10.1 (1, 26.2) | 2.4 (0.9, 26.4) |

^a^Based on Kaplan–Meier product-limit method; ^b^Log-rank test.

CI, confidence interval; KM, Kaplan–Meier; NA, not available; QMG Quantitative Myasthenia Gravis; SD, standard deviation.
